# The impact of γ-irradiation on the induction of bystander killing by genetically engineered ovarian tumor cells: implications for clinical use

**DOI:** 10.1186/s12935-017-0465-z

**Published:** 2017-10-24

**Authors:** Jehad Zweiri, Stephen E. Christmas

**Affiliations:** 1 Department of Molecular and Haematological Medicine, King’s College London, The Rayne Institute, 123 Coldharbour Lane, London, SE5 9NU UK; 2 Institute of Infection & Global Health, University of Liverpool, Liverpool, UK; 3 Dept. of Clinical Infection & Immunology, Institute of Infection & Global Health, Ronald Ross Building, University of Liverpool, 8, West Derby St, Liverpool, L69 7BE UK

**Keywords:** Tumor cell lines, γ-irradiation, Suicide gene therapy, Anti-tumor immune response, Cell death, Bystander killing effect, Cancer clinical trials

## Abstract

**Background:**

Cellular based therapeutic approaches for cancer rely on careful consideration of finding the optimal cell to execute the cellular goal of cancer treatment. Cell lines and primary cell cultures have been used in some studies to compare the in vitro and in vivo efficacy of autologous vs allogeneic tumour cell vaccines.

**Methods:**

This study examines the effect of γ-irradiation on a range of tumor cell lines in conjunction with suicide gene therapy of cancer. To determine the efficacy of this modality, a series of in vitro and in vivo experiments were conducted using genetically modified and unmodified tumor cell lines.

**Results:**

Following co-culture of HSV-TK modified tumor cells and unmodified tumor cells both in vitro and in vivo we observed that the PA-STK ovarian tumor cells were sensitive to γ-irradiation, completely abolishing their ability to induce bystander killing of unmodified tumor cells. In contrast, TK-modified human and mouse mesothelioma cells were found to retain their in vitro and in vivo bystander killing effect after γ-irradiation. Morphological evidence was consistent with the death of PA-STK cells being by pyknosis after γ-irradiation. These results suggest that PA-STK cells are not suitable for clinical application of suicide gene therapy of cancer, as lethal γ-irradiation (100 Gy) interferes with their bystander killing activity. However, the human mesothelioma cell line CRL-5830-TK retained its bystander killing potential after exposure to similarly lethal γ-irradiation (100 Gy). CRL-5830 may therefore be a suitable vehicle for HSV-TK suicide gene therapy.

**Conclusions:**

This study highlights the diversity among tumor cell lines and the careful considerations needed to find the optimal tumor cell line for this type of suicide gene therapy of cancer.

## Introduction

The central objective in cancer therapy is to kill the malignant cells while causing little or preferably no collateral damage to healthy cells. Suicide gene therapy, as applied to the treatment of cancer, holds the potential to achieve just that [1, 2]. An example is the insertion of the herpes simplex virus thymidine kinase (HSV-TK) gene into cancer cells which are consequently induced to “commit suicide” when in the presence of otherwise non-toxic doses of ganciclovir (GCV) [3, 4]. This selective toxic effect of the purine analogue ganciclovir is because HSV-TK phosphorylates ganciclovir, converting it ultimately to ganciclovir-triphosphate, a toxic compound when inserted into the DNA of these transfected cells [5–7]. As with any other gene therapy strategy, and any anti-cancer treatment approach, its main limitation is the selective targeting and transduction of all tumor cells in vivo [8]. However, it may not be necessary to transduce every tumor cell in vivo to bring about a clinically-meaningful anti-tumor effect [9–11]. Indeed, it has been demonstrated that two types of “bystander tumor cell killing” mechanisms are mediated by this approach: (a) a “direct” bystander effect, due to the transfer of ganciclovir triphosphate from HSV-TK-positive tumor cells into untransfected neighboring cells [12–14], (b) a systemic immunologically-mediated bystander effect due to the in vivo immune presentation of tumor-specific/associated antigens following the killing of HSV-TK—expressing cells [15, 16].

The genetically modified HSV-TK human ovarian cancer cell line, PA1-STK, has been used for the treatment of solid tumors (administered intraperitoneally in patients with ovarian cancer) [17]. The in vitro culture of these cells in the presence of ganciclovir provoked bystander killing, but with limited cytotoxic activity in vivo [18]. The rationale for this strategy was that PA-STK cells, injected in the vicinity of the patient’s tumor bulk, could make contact with, and seed themselves onto, the patient’s tumor cells in vivo and, after treatment with ganciclovir, could commit suicide and kill the patient’s tumor cells by a “direct” bystander mechanism (e.g. gap junction mediated transfer of the phosphorylated ganciclovir from the HSV-TK positive cells to the TK negative cells. This direct cytotoxicity could then induce a more systemic immunological bystander tumor-killing effect [19–21].

Irradiation in conjunction with cancer gene therapy may have several potential benefits. As well as synergistic killing of tumor cells, it may enhance the bystander effect by releasing tumor antigens, stimulating a broad anti-tumor immune response [19–22]. However, it is important to establish that irradiated HSV-TK-containing cells are still able to induce direct bystander killing, without contributing to the live tumor burden. This is a particularly important consideration when such treatment is considered for conditions of low tumor cell burden, but poor prognosis, such as surgically debulked advanced malignancies.

The objective of the present studies was to assess the efficacy of bystander killing of unmodified tumour cells by HSV-TK modified cells, before and after irradiation. The studies reported here show that γ-irradiation has a vastly different impact on the viability of tumor cells in vitro; some die within hours whereas others remain metabolically active for several days. The consequences of such different sensitivities to γ-irradiation are that the highly radiosensitive cells failed to mediate efficient bystander killing either in vitro or in vivo, whilst the less radiosensitive cells were able to mediate efficient bystander killing both in vitro and in vivo. These studies highlight the importance of sensitivity to γ-irradiation for the development of allogeneic cell lines for the clinical application of HSV-TK mediated bystander killing of tumor cells.

## Methods

### Cell lines

Human mesothelioma cell lines, CRL-5915, 5820, and 5830 were obtained from the American type culture collection (Rockville, MD, USA) with the permission of Prof. A Gazdar (MD Anderson Cancer Center, Texas, USA) [23]. Mouse mesothelioma cell lines, ABI (H-2d) from BALB/c mice, AE17 (H-2k) from CBA mice, and AC29 (H-2b) from C57BL/6 mice were obtained from Prof. B. Robinson, QEII Medical Centre, University of Western Australia, Nedlands, Australia [24]. Human ovarian tumor (teratocarcinoma) cell line, PA-1 and PA-STK were obtained from Prof. S Freeman, Tulane University Medical School, New Orleans, USA [17]. Three ovarian tumor cell lines already available in the laboratory were: OVC-432 (ovarian carcinoma, a kind gift from Dr C. Dohring, Basel Institute for Immunology, Basel, Switzerland [25], SKOV-3 (ovarian adenocarcinoma) [26], and OIB, an ovarian tumor cell line established by Prof. F Farzaneh in the Molecular Medicine Department [27]. All human cell lines were maintained in DMEM, supplemented with 10% Fetal Calf Serum (FCS) and 1% sodium pyruvate. In the case of mouse mesothelioma cells, the RPMI-medium was supplemented with 5% FCS, 20 mM HEPES, 50 mM 2-mercaptoethanol (2ME) and 2 mM glutamine.

### Retroviral infection

Cells to be infected were seeded at a density of 5 × 10^5^ cells per 10 cm plate, 24 h before infection. Virus producing cells were also grown to about 90% confluence, and the medium changed 10 h prior to harvest of the culture medium to ensure that fresh virus containing supernatant was used [28].

The medium from virus producing cells was removed and filtered through a 0.45 μm pore-size filter to remove cell debris but allowing passage of the viral vector through the filter. To enhance the retroviral infection, 8 μg/ml of polybrene (Sigma Aldrich, Poole, UK) was added to the culture medium. Polybrene is required for coating of target cells in order to neutralise their negative surface charge, therefore increasing the efficiency of infection [29, 30]. The medium from the cells to be infected was removed prior to infection, and the vector-polybrene mixture added. After 10 h of infection the medium was changed. 48 h after infection, the medium was removed and replaced with fresh medium containing G418 at 1 mg/ml. G418 resistant clones were expanded.

### In vitro GCV-sensitivity studies on the tumor cell lines

The in vitro studies examining the effect of GCV on the HSV-TK expressing mesothelioma and ovarian carcinoma cells, as well as the untransduced cells, were performed in 96-well plates. Transduced and untransduced cells were plated in triplicate at two densities, 10^4^ cells/well and 10^5^ cells/well for comparison. After 2 days, the medium was replaced by fresh DMEM containing the indicated concentrations of GCV in the range 10,000–0.01 μM. Cells were then incubated at 37 °C in humidified 5% CO_2_ for 5 days. Sensitivity to GCV treatment was measured by using a colorimetric cell proliferation assay that measures viable cell dehydrogenase activity, the microculture tetrazolium cell proliferation assay [31]. 20 μl of 5 mg/ml MTT (Sigma Aldrich, Poole, UK) was added to each well for 3 h and the cells in each well were solubilised in 150 μl of MTT solubilisation solution. After overnight incubation, the optical density of each well was measured on a 96-well plate reader (Dynatech, Reading, UK) set at 570 nm wavelength. Known concentrations of cells were also plated, cultured in the presence of MTT, and similarly solubilized. The absorbance reading of these control cells represents the metabolic activity of a known number of cells and was used to generate a standard curve in Microsoft Excel. The absorbance reading for each sample (well) was directly compared with the standard curve, and the numbers of viable cells were determined accordingly.

### Bystander killing effect studies

The bystander effect was determined by mixing HSV-TK expressing cells with untransfected cells at the indicated ratios. Cells were then plated in triplicate in 10 cm plates at two densities, 1 × 10^5^ and 5 × 10^5^ cell/plate to ensure cell–cell contact and to compare the in vitro effect of cell densities on the bystander effect. Two days later, the cells were treated with 50 μM GCV and incubated at 37 °C, 5% CO_2_ for 10–14 days. The plates were then stained with 2% methylene blue and stained cells were counted.

In order to calculate the effect of GCV on the mixed PA-STK and PA-1 populations, the number of colonies counted were expressed as a percentage of the total number of colonies in the co-cultured TK + ve and TK − ve tumor cells at the indicated ratios of the two cell populations in the presence of the indicated concentrations of GCV. A graph was obtained by plotting the percentage of surviving colonies.

### γ-irradiation of HSV-TK modified tumor cells

2 expressed as 10^6^ tumor cells resuspended in 5 ml of DMEM were γ-irradiated (100 Gy) using a Gamma cell-1000 (Atomic Energy of Canada Ltd. Source: ^137^Cs).

### Cytospin histological analysis

A 100 μl aliquot of 10^4^ cells/ml in PBS was used to prepare cytospin slides using a cytospin system (Hettich, Salford, UK), fixed in 10% formalin for 10 min, left overnight to air-dry and then stained by the Papanicolaou staining method [32, 33].

### Measurement of apoptosis and necrosis by FACS analysis

Tumor cells were resuspended in 100 μl of working labelling solution and incubated for 15 min in the dark. Two control tubes were used: cells stained with Annexin V-FITC (Becton–Dickinson, Oxford, UK) alone and cells stained with PI alone [34, 35]. The cells were then analysed by flow cytometry using a FACScan analyser (Becton–Dickinson, Oxford, UK).

### Cell cycle analysis

Cells were irradiated at 100 Gy and incubated overnight at 37 °C, in 5% CO_2_, resuspended in 1 ml of staining buffer (0.1% sodium citrate, 0.1% Triton-X100 and 10 μg/ml propidium iodide in dH_2_O), vortexed and left overnight at 4 °C in the dark. The cells were then analysed by flow cytometry using a FACScan analyser.

### In vivo studies: inoculation, establishment and treatment of the tumors intraperitoneally (IP) in mice

AB1 mouse mesothelioma adherent tumor cells were harvested by washing with versene only and re-suspended in 0.2 ml of PBS. Tumors were established IP in female BALB/c mice by injecting the mesothelioma cells (AB1 tumor cell line), at different doses (1 × 10^5^, 5 × 10^5^ and 1 × 10^6^/100 ml PBS) using a 26-gauge needle, to establish the TD_50_ for the tumor cell line, according to Home Office regulations. Mice (n = 45) were injected i.p. with AB1 tumor cells on day 0. Nine days later, animals were assigned to nine groups (n = 5 per group). GCV treatment was started at day 10. GCV (Cymevene® 500 mg; Roche, Switzerland) was diluted in sterile DMEM to a stock concentration of 50 mg/ml. The stock solution of GCV was diluted in DMEM to a concentration of 2 mg/ml, and 1 ml of the stock was injected IP once a day for 5 consecutive days. Mice were monitored every 2 days to palpate the tumor. At post-mortem, all tumor nodules were counted and measured using a calliper (for each nodule, 2 perpendicular diameters were recorded). Tumor volume was calculated for each nodule assuming spherical shape and the total tumor volume was calculated by adding all the calculated values for each mouse.

### Statistical analysis

Statistical analysis was performed using the Microsoft Excel program. Differences between groups were analysed using Student’s paired *t* test. A P value of < 0.05 was considered as significant.

## Results

### PA-STK cells irradiated at 100 Gy lose their ability to induce bystander killing

**Fig. 1:**
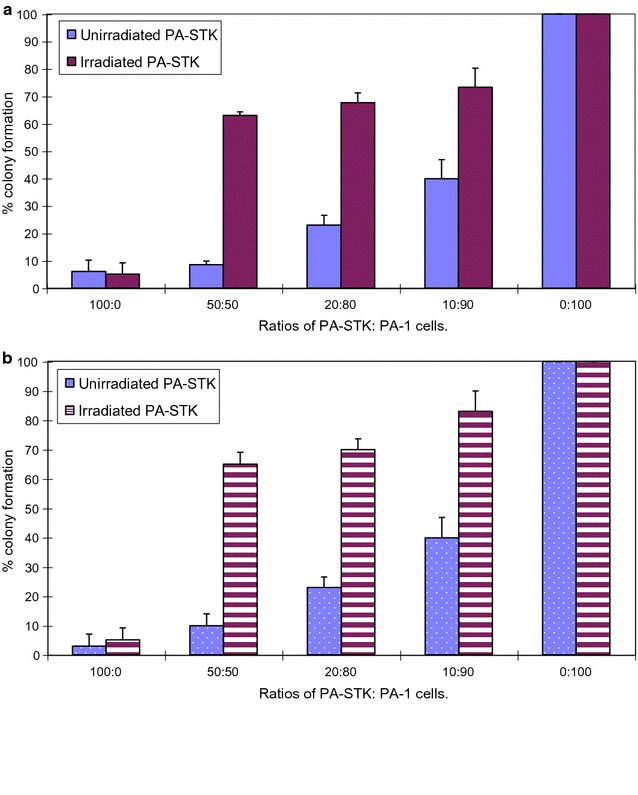
Loss of bystander killing after γ-irradiation of PA-STK cells (100 Gy) **a** 5 × 10^5^ cells/plate; **b** 2 × 10^6^ cells/plate. Mixing experiments demonstrate the in vitro bystander effect of the irradiated and un-irradiated PA-STK cells. X-axis represents the ratios of PA-STK to PA-1 cells. Y-axis represents % colony formation after exposure to 50 μM GCV for 5-days. Error bars represent standard error of the mean. Representative of three similar experiments

Irradiation of PA-STK cells (100 Gy) substantially reduced their ability to induce bystander killing of unmodified PA-1 cells (Fig. 1a). This study was carried out at the optimum number of 5 × 10^5^ cells per 10 cm^3^ plate as previously determined (data not shown). A possibility was that the irradiation stopped the growth of PA-STK cells and therefore reduced the possibility of cell to cell contact in the tissue culture plate. This experiment was therefore repeated at a higher cell density of 2 × 10^6^ cells/plate. Increasing the cell density did not restore the bystander effect (Fig. 1b). At both cell densities, irradiated PA-STK cells were highly significantly less efficient at mediating the bystander effect at a 50:50 ratio than unirradiated cells (P = 0.04). Similar data were obtained in three further repeats of this experiment.

### γ-Irradiated (100 Gy) human and mouse mesothelioma cells retain the ability to induce bystander killing

**Fig. 2:**
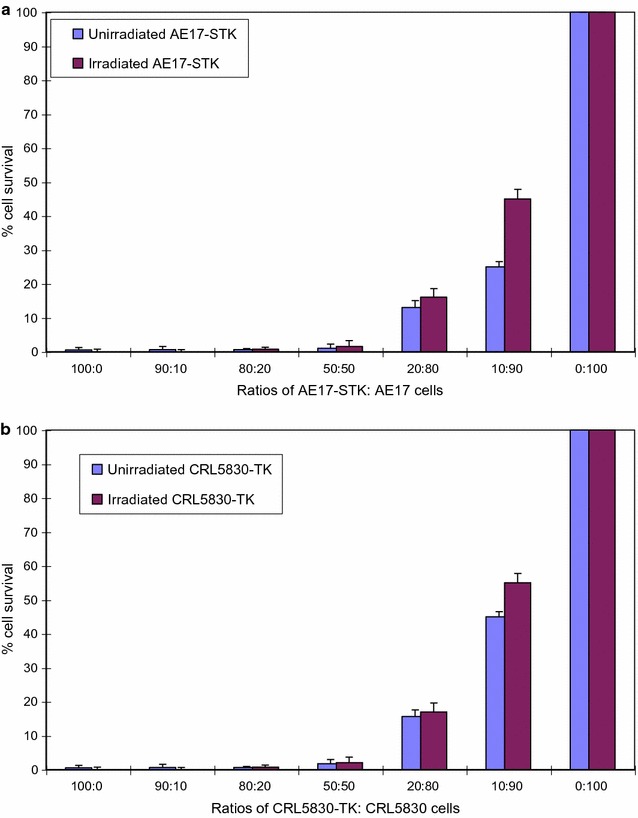
In vitro bystander killing induced by γ-irradiated (100 Gy) mouse mesothelioma AE17-STK cells. **a** AE17-STK and AE-17 cells (with or without γ-irradiation) were mixed and cultured in the presence of 50 μM GCV for 6 days. The total number of cells was 5 × 10^4^/well of 96-well tissue culture plate. Per cent survival was measured using the MTT assay. Each point is the mean of three separate measurements and error bars indicating the standard error of the mean are shown. Similar data was obtained in three separate experiments. **b** In vitro bystander killing induced by the γ-irradiated human mesothelioma cell line CRL-5830TK. CRL5830-STK and CRL5830 cells were mixed in the indicated ratios and cultured in the presence of 50 μM GCV for 6 days. The total number of cells was 5 × 10^4^/well of 96-well tissue culture plate. The fraction of surviving cells was measured using the MTT assay. Each point is the mean of three separate measurements and error bars indicate the standard error of the mean. Similar data was obtained in three separate experiments

In contrast to the PA-STK cells, the mouse mesothelioma AE17-STK cells retain their capacity to induce bystander killing after γ-irradiation (100 Gy, Fig. 2a). Human mesothelioma cells CRL-5830-TK, were similarly able to retain their bystander killing activity after γ-irradiation (Fig. 2b). In neither case was there a significant difference in efficacy between irradiated and unirradiated cells (P > 0.45 at all cell ratios).

### Investigation of PA-STK cell death after irradiation

**Fig. 3:**
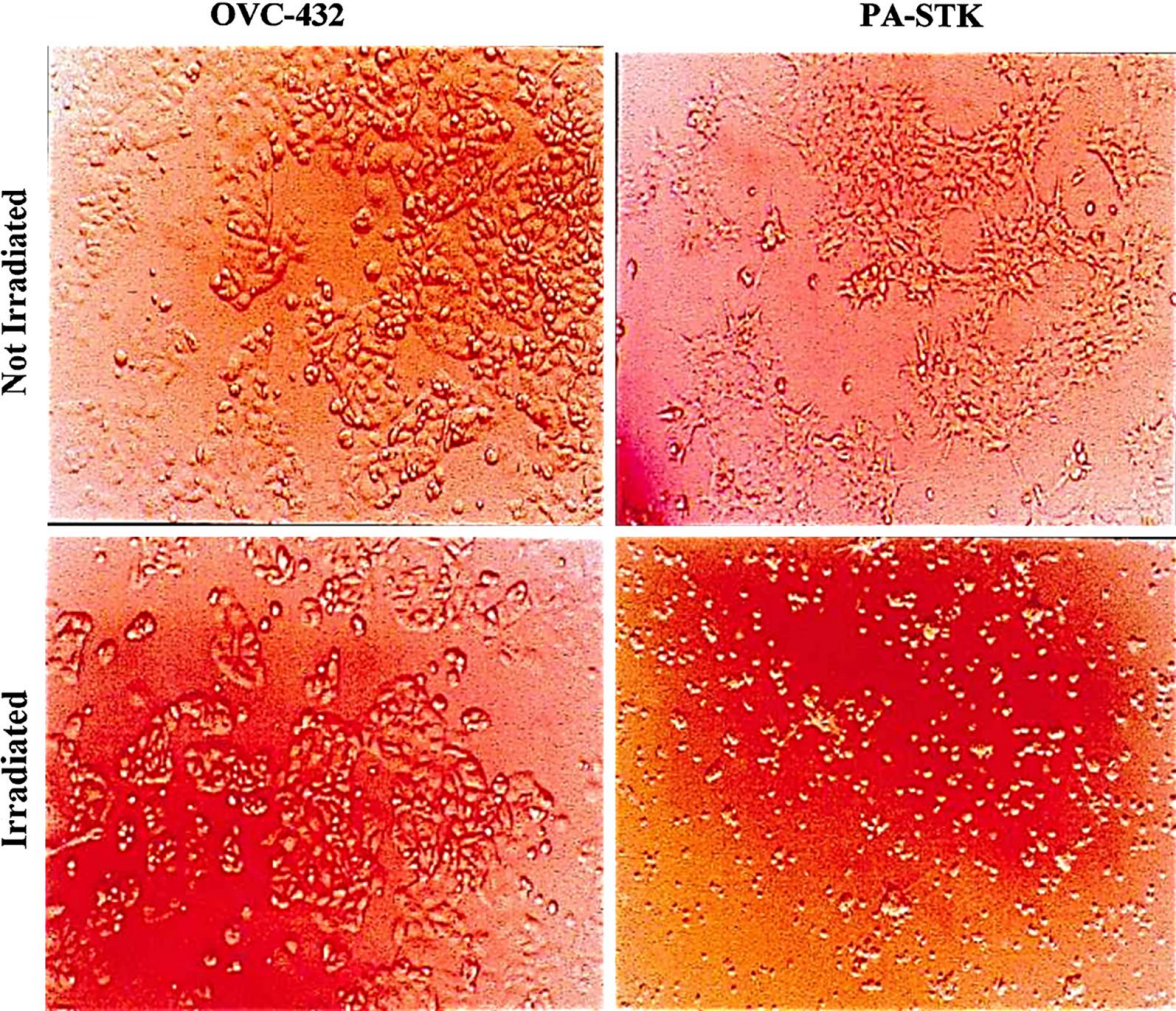
The effect of γ-irradiation on OVC-432, PA-1, and PA-STK cells. The cells were either γ-irradiated at 100 Gy, or not irradiated as controls. The cells were re-plated in 6-well tissue culture plates at a density of 5 × 10^4^ per plate and then were incubated overnight. The plates were then visualized and photographed by light microscopy (100 × magnification)

Microscopic examination of the irradiated (100 Gy) PA-STK and PA-1 cells revealed that they were very sensitive to irradiation. The cells did not attach to the culture plate after irradiation, when visualised the next day (Fig. 3). In contrast, OVC-432 cells were able to efficiently adhere to the culture dish, even after 100 Gy γ-irradiation. These cells, in common with all other cell lines tested (OVC-432, SKOV-3, OIB, CRL-5830, CRL-5839-STK, CRL-5820, CRL-5915, AE17, AE-STK, AB-1, AC-29, HL-60) were able to attach efficiently to substrate and remain metabolically active for about 3 days before eventually losing surface adherence (data not shown).

A dose range of irradiation from 1 to 100 Gy was then tested on PA-STK cells. For this purpose the cells were trypsinised, γ-irradiated and then plated onto tissue culture plates. The next day it was observed that even the PA-STK cells irradiated at 1 Gy had lost their ability to adhere to the tissue culture plates. Further cell viability studies were performed using the MTT assay to determine their viability, showing that these cells were non-viable at all γ-irradiation doses. Moreover, these cells failed to mediate any in vitro bystander effect at all γ-irradiation doses tested (data not shown).

### Analysis of cell death in PA-STK cells

**Fig. 4:**
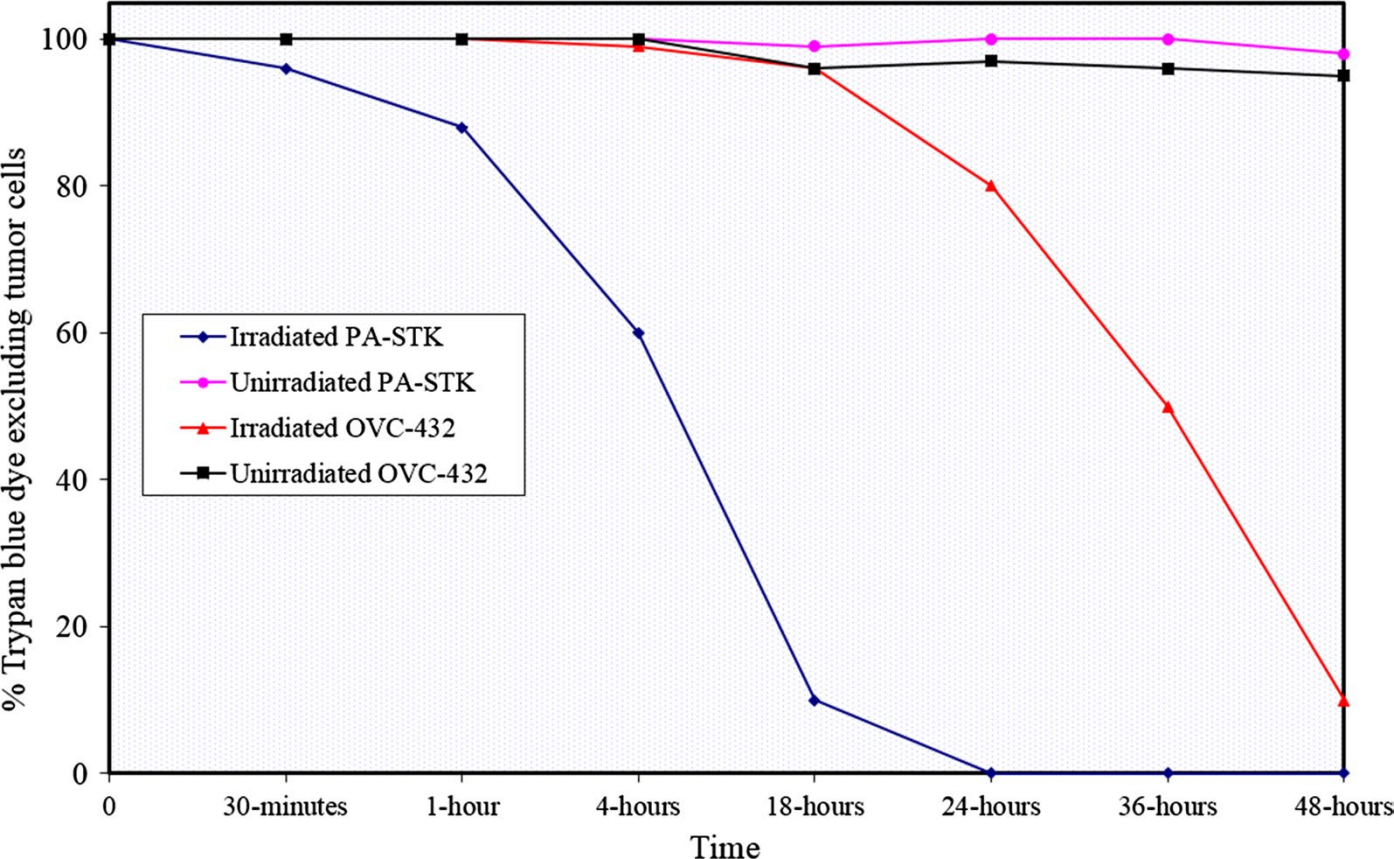
Time course of cell death in γ-irradiated PA-STK and OVC-432 cells using trypan blue dye exclusion. The cells were irradiated at 100 Gy and the percentage of trypan blue excluding cells was determined at the indicated time points. The counts were performed in triplicate. Error bars represent standard error of the mean. Representative of three similar experiments

The first objective was to quantitatively record cell death during a time course study following γ-irradiation using the trypan blue dye exclusion method (Fig. 4). The control cell line used in this experiment was another ovarian cancer cell line, OVC-432, as well as non-irradiated PA-STK cells. The vast majority (over 90%) of the irradiated PA-STK cells died by 18 h post irradiation. However, this was not the case for control cells which survived beyond 48 h.

**Fig. 5:**
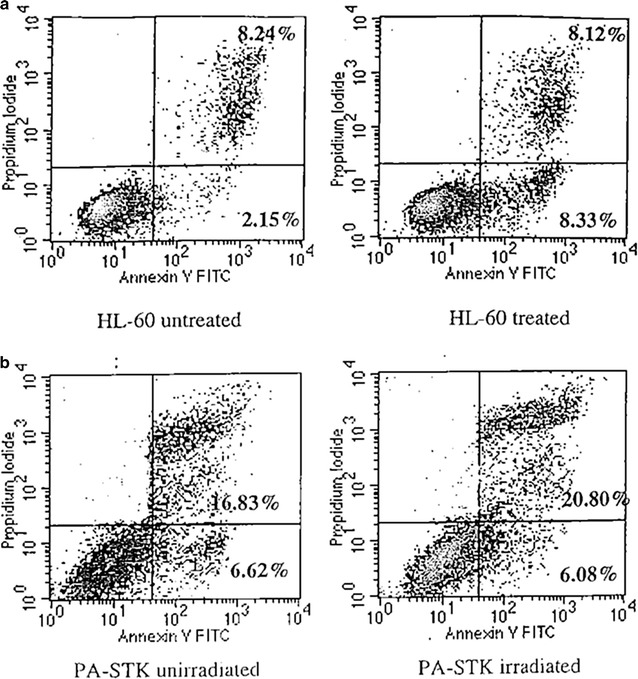
Apoptosis measured in two human tumor cell lines using annexin V/propidium iodide. Cells were analysed with a FACSCalibur flow cytometer: **a** HL-60 control cell line either untreated or treated with 0.1 µM staurosporin for 4 h. **b** PA-STK cells unirradiated or irradiated at 100 Gy. The irradiated tumour cells were analysed 10 h after irradiation

Additionally, Annexin-V and propidium iodide staining of cells was used as a method to quantify the proportion of apoptotic and necrotic cells in the γ-irradiated population of PA-STK cells, respectively. In this experiment HL-60 cells treated with staurosporine (1 mg/ml for 4 h) were used as positive control cells [34, 35]. As shown in Fig. 5, only a small proportion of PA-STK cells were dying by apoptosis.

**Fig. 6:**
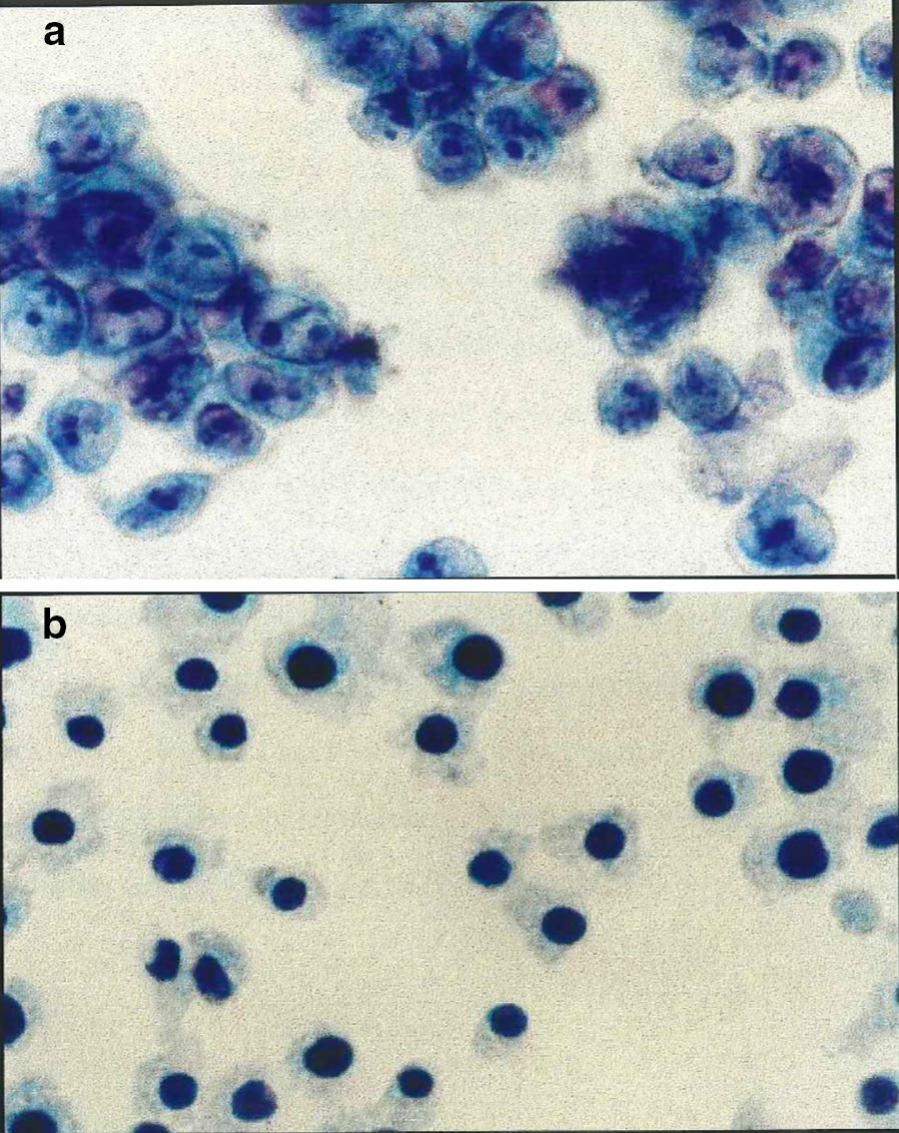
Cytospin-histological analysis of irradiated PA-STK ovarian tumor cells. **a** control un- irradiated cells. **b** The PA-STK cells γ-irradiated at 100 Gy, after 18 h in culture 1 × 10^4^ cells were centrifuged at 2400 rpm for 10 min. Papanicolaou staining was then performed and cells observed at 1000 × magnification

In order to further examine the mechanism of cell death in the irradiated PA-STK cells, cytospin preparations were examined 18 h later. The non-irradiated PA-STK cells served as controls. The cytospin histological analysis of the irradiated PA-STK cells suggests that these cells die by pyknosis, a form of necrosis (Fig. 6). Pyknosis is characterised by nuclear shrinkage and increased basophilia [36]. In the irradiated PA-STK cells the DNA condensed into a solid, shrunken basophilic mass. The only clear inference which can be drawn from these data is that the radiation induced death in the vast majority of PA-STK cells is due to necrosis/pyknosis, rather than apoptosis.

### In vivo bystander killing activity

**Fig. 7:**
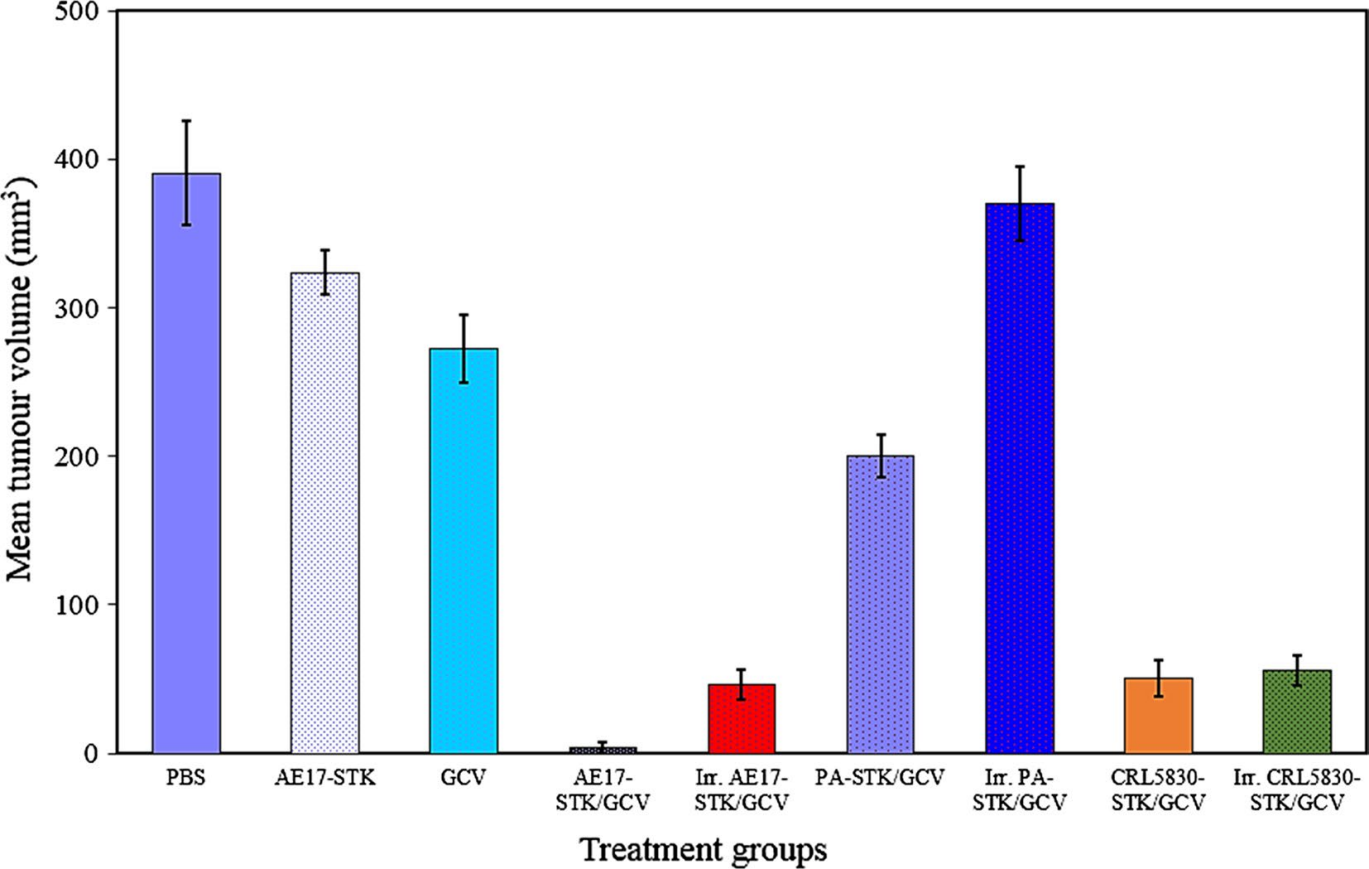
In vivo treatment using allogeneic AE17-STK mouse mesothelioma cells in BALB/c mice. BALB/c mice were injected IP with the syngeneic murine mesothelioma cells AB1 (1 × 10^6^ cells per mouse) on day 0. Nine days later the mice were separated into nine treatment groups as indicated, and another healthy group acted as positive control. On day 18, mice were sacrificed and post-mortem was performed. All tumor nodules were counted and measured using a calliper. Tumor volume was calculated for each mouse and the mean tumor volume was calculated for each group. The error bars represent standard error of the mean. Irr. Stands for cells irradiated at 100 Gy and treated with GCV. See text for detailed experimental conditions. Error bars represent standard error of the mean. Representative of three similar experiments

To establish an allogeneic TK-tumor cell/GCV treatment model, a series of experiments were conducted to repeat and extend previous in vivo studies. The treatment groups in Fig. 6 were used to assess and compare the activity of PA-STK cells (irradiated and non-irradiated) as a possible vehicle for HSV-TK suicide gene therapy with other TK expressing tumor cells. In this experiment BALB/c mice were injected with the syngeneic murine AB1 mesothelioma cells intraperitoneally (1 × 10^6^ cells per mouse) on day 0 (to act as the experimental groups), and one group of mice acted as healthy controls. Nine days later, the mice were separated into 9 different treatment groups. Group 1: PBS alone intraperitoneally (2 ml), Group 2: AE17-STK alone (2 ml containing 1 × 10^6^ cells), Group 3: GCV alone (2 mg/ml/mouse for 5 consecutive days), Group 4: GCV + AE17-STK (GCV 2 mg/mouse for five consecutive days), Group 5: γ-irradiated AE17-STK/GCV (100 Gy) + GCV, Group 6: unirradiated PA-STK cells + GCV, Group 7: γ-Irradiated PA-STK (100 Gy) + GCV, Group 8: CRL5830-STK/GCV, and Group 9: γ-Irradiated CRL5830-STK/GCV (100 Gy). Co-cultured cells for treatment were used at 50% HSVTK modified tumor cells: 50% unmodified tumor cells. The mice were then left and monitored for signs of tumor development and toxicity. Eight days after the first day of treatment (i.e. 17 days from tumor inoculation), mice were sacrificed and post-mortem was performed. The mean tumor volume was calculated for each treatment group (Fig. 7).

Despite the toxicity of the treatment, the animals in Group 4 (which received AE17-STK + GCV) had significantly smaller tumors compared to the mice in the first 3 control groups (PBS alone, AE17-STK alone, or GCV alone) [P = 0.03]. This reduction in tumor volume appeared to be reduced slightly in the case of irradiated AE17-STK cells, as there was a smaller reduction in tumor volume in Group 5 (which received γ-irradiated AE17-STK with GCV) compared to Group 4 (GCV + AE17-STK), although this reduction was not statistically significant (P = 0.56). For Groups 6 and 7 (PA-STK treated groups), the unirradiated PA-STK cells + GCV mediated a detectable anti-tumor effect in vivo, whereas the γ-irradiated PA-STK cells and GCV did not (P values = 0.50 and 0.6 respectively), compared to control mice which received PBS only. Moreover, both the live and irradiated CRL5830-STK human mesothelioma cells in combination with GCV mediated a detectable anti-tumor bystander killing effect, compared to control mice which received PBS only, P values = 0.05 and 0.06 respectively.

## Discussion

The use of lethally-irradiated allogeneic TK-modified tumor cells could result in the development of a ‘generic’ HSV-TK expressing tumor cell line to generate an anti-tumor immune response against a number of different tumor types [17, 37]. If effective, such a strategy could be much more practical, as it could be more easily standardised. In addition it could be a less expensive therapeutic option than therapy with autologous modified tumor cells [38].

The studies described above appear to indicate that irradiation of PA-STK cells abolishes their bystander killing ability, because the PA-STK cells are very radiosensitive and die too rapidly to enable therapeutic benefit. The radio-sensitivity of the PA-1 and PA-STK cells appeared to be much higher than a panel of 10 other cell lines including human and mouse mesothelioma cells. The precise mechanism for this difference is unclear but could be a result of a deficiency in DNA repair in PA-STK cells.

In a murine mesothelioma model, Schwarzenberger and his colleagues successfully used PA-STK ovarian tumor cells to treat peritoneally grown mesothelioma tumor AC29. In this study the PA-STK cells were not irradiated [18]. Efficacy of the irradiated TK + ve cells was tested in a previous study by Freeman and his colleagues using the TK + ve cells KBALB but not PA-STK cells. However, the data presented in this study demonstrate that irradiation of PA-STK cells, even at very low doses, makes them lose their capacity to induce an in vitro bystander effect as they die very early post-irradiation.

In this study, irradiated and non-irradiated AE17-STK mouse mesothelioma cells were examined for their efficiency in inducing the rejection of allogeneic tumors; AE17-STK cells were also compared with a human mesothelioma cell line CRL5830. Tumor regression was detected in all animals injected with the non-irradiated allogeneic AE17-STK cells after GCV administration whereas in mice treated with the irradiated cells tumor regression was detectable in only 60% of the animals. Interestingly, the human mesothelioma CRL5830-STK in combination with GCV treatment has indicated significant reduction in tumor mass in contrast to PA-STK human cell line.

The first clinical trial to use a suicide gene (HSV-TK) as a primary therapeutic agent was approved in 1991 for the treatment of ovarian carcinoma [17]. Ovarian cancer cells (PA-1) were transfected ex vivo with the gene for HSV-TK and then injected intraperitoneally in nine patients with stage III disease. One patient achieved complete remission, whereas others showed partial tumor regression. However, data obtained in the present study show that these particular cells (PA-STK) were not able to mediate any detectable in vivo bystander killing after lethal irradiation; however a detectable but not significant bystander killing was found when these cells were not irradiated. The high sensitivity of PA-STK to irradiation appeared to be responsible for the complete loss of the bystander effect not only in vivo but also in vitro. The non-irradiated PA-STK cells were able to mediate bystander killing in vitro, and also to induce a detectable level of in vivo bystander killing. One possible explanation is that the PA-STK cells are not very efficient mediators of the direct bystander effect (PA-STK IC50 of 20 mM GCV, compared to an IC50 of 3 mM for AE17-STK or 1 mM for CRL5830-TK cells). It is possible that the xenogeneic human ovarian PA-STK cells did not very efficiently home onto and/or establish gap junctions with the allogeneic murine mesothelioma cells [10, 39]. However, as already mentioned, Schwarzenberger and colleagues did find that PA-STK cells can treat AC29 mouse mesothelioma tumors initiated 4 days earlier [18]. However, in the present studies in which the PA-STK cells were given on day 9 of tumor inoculation there was no evidence of tumor regression after irradiation, although there was clear evidence for the induction of tumor elimination by the AE17-STK cells.

To sum up, the issue of tumor cell sensitivity to γ-irradiation is critical in cellular therapy, as ‘optimum’ sensitivity need to be observed carefully. Gene-modified (HSVTK) tumor cells which are to be used in cancer patients need to be lethally irradiated so that they will not proliferate in the recipient’s body, but still be able to mediate bystander killing. It is therefore important to establish whether and when the irradiated gene-modified tumor cells would die after irradiation.

## Conclusions

This study suggests that PA-STK ovarian tumor cells are rather poor mediators of bystander killing, and therefore may not be the most efficient cells for future clinical trials. However, the in vitro and in vivo results obtained in the present studies suggest that CRL-5830TK human mesothelioma cells may be effective inducers of bystander killing for the clinical treatment of mesothelioma. Interestingly, CRL-5830TK cells appear to retain their killing ability even after lethal irradiation.

Although encouraging results are emerging from pre-clinical and some early stage clinical studies, further developments are needed. These include: (1) development of relevant disease models in higher vertebrates with physiological traits that are adequately representative of humans malignancies; (2) development and optimisation of the gene delivery systems; (3) further investigation of ways of enhancing the bystander effect; (4) modulation of the host immune response to enhance and complement suicide gene therapy; (5) further exploration of other therapeutic strategies in combination with suicide gene therapy in the light of the observation that certain suicide genes can behave as radio-sensitising agents [40–42].

## Abbreviations

GCV: ganciclovir
HSV-TK: herpes simplex virus thymidine kinase
MTT: micro-culture tetrazolium cell proliferation assay
